# The L-shaped association of mid-upper arm circumference with all-cause and cause-specific mortality in US adults: a population-based prospective cohort study

**DOI:** 10.1186/s12889-023-17064-x

**Published:** 2023-11-20

**Authors:** Xinran Hou, Jie Hu, E. Wang, Qulian Guo, Xian Zhang, Minjing Yang, Zhuoyi Liu, Jian Wang, Zongbin Song

**Affiliations:** 1 Department of Anesthesiology, Xiangya Hospital, Central South University, Changsha, China; 2 National Clinical Research Center for Geriatric Disorders (Xiangya Hospital), Central South University, Changsha, China; 3 Department of Occupational and Environmental Health, Xiangya School of Public Health, Central South University, Changsha, China

**Keywords:** Mid-upper arm circumference, All-cause mortality, Cardiovascular disease, Cancer, Respiratory disease, NHANES

## Abstract

**Background:**

The arm circumference is a feasible and reliable indicator in evaluating the nutritional status of children. However, its application in general adults has yet to be thoroughly investigated.

**Objective:**

This study aimed to evaluate the association between mid-upper arm circumferences (MUACs) and mortality in general adults.

**Methods:**

The nationally representative cohort from the National Health and Nutrition Examination Survey (1999—2018) was analyzed with mortality information obtained through linkage to the National Death Index. The baseline MUACs were collected as exposure. Survey-weighted Cox proportional hazard regressions were performed to estimate the hazard ratios (HRs) and 95% confidential intervals (CIs) of mortality risk for individuals with different MUACs. Restricted cubic spline analyses were performed to examine the nonlinear association of MUAC with all-cause and cause-specific mortality.

**Results:**

A total of 52,159 participants were included in this study. During a median follow-up time of 117 months, 7157 deaths were documented, with leading causes of cardiovascular disease (CVD), cancer, and respiratory disease. Individuals in the first quartile (Q1) of MUAC tended to have higher all-cause mortality risk than the rest after full adjustment. Similarly, CVD mortality risk in Q1 was higher than that in the second quartile (Q2) and the third quartile (Q3); respiratory mortality risk in Q1 was higher than in Q2. MUAC was non-linearly associated with all-cause mortality and CVD mortality. Individuals in Q1 MUAC (≤ 29.3) tended to have higher all-cause mortality risk, with HRs (95% CIs) estimated to be 0.76 (0.67–0.87) for Q2 (29.4, 32.5), 0.69 (0.59–0.81) for Q3 (32.6, 36.0), and 0.59 (0.46–0.75) for Q4 (≥ 36.1) after adjustment of demographic, lifestyle, and comorbidity covariates. Similarly, compared with Q1, HRs (95% CIs) for CVD mortality were estimated to be 0.73 (0.58–0.93) for Q2 and 0.57 (0.43–0.47) for Q3; HRs (95% CIs) for respiratory mortality was estimated to be 0.57 (95% CI, 0.37–0.87) for Q2 with other differences not significant.

**Conclusion:**

The MUAC was inversely associated with long-term mortality in general adults in the United States and may serve as a valuable measurement in adult health evaluations.

**Supplementary Information:**

The online version contains supplementary material available at 10.1186/s12889-023-17064-x.

## Introduction

Arm circumference, represented by mid-upper arm circumference (MUAC) in this study, is a cost-effective and straightforward anthropometric measure that refers to the rim of the upper arm measured at the midpoint between the tip of the elbow (olecranon process) and that of the shoulder blade (acromion). MUAC has been widely used to evaluate the nutritional status of infants and children [1–3] and to assess child mortality risks associated with malnutrition [4, 5]. However, due to insufficient recognition of its relationship with long-term health outcomes, the application of MUAC in adult health evaluation has yet to be universally acknowledged.

In recent years, low arm circumference has been associated with increased mortality risk in older individuals [6–8], cirrhosis patients [9], heart failure patients [10], and hemodialysis patients [11]. However, the association of arm circumference with mortality in the large-scale general population has yet to be thoroughly investigated, and substantial divergences have been reported [12, 13].

We hypothesized that arm circumference could also act as a health indicator in the general adult population, in which adults with low arm circumference may suffer from high risks of premature death. Thus, in the present study, we intended to explore the association of MUAC with all-cause mortality and cause-specific mortality based on a national representative cohort from the continuous National Health and Nutrition Examination Survey (NHANES) 1999 to 2018 dataset with linkage to the National Death Index (NDI) mortality files. Our results might provide some evidence for the potential role of MUAC in adult health evaluation by its association with long-term health outcomes.

## Methods

### Study design and participant selection

The NHANES is a continuous program conducted by the National Center for Health Statistics (NCHS) of the Centers for Disease Control and Prevention. It examines a nationally representative sample of approximately 5,000 participants each year from the noninstitutionalized US population. The protocols of NHANES have been approved by the NCHS ethics review board, and written informed consents were obtained from all participants. This study analyzed deidentified publicly available data and followed the Strengthening the Reporting of Observational Studies in Epidemiology (STROBE) reporting guidelines for cohort studies [14] (Table S1).

**Fig. 1:**
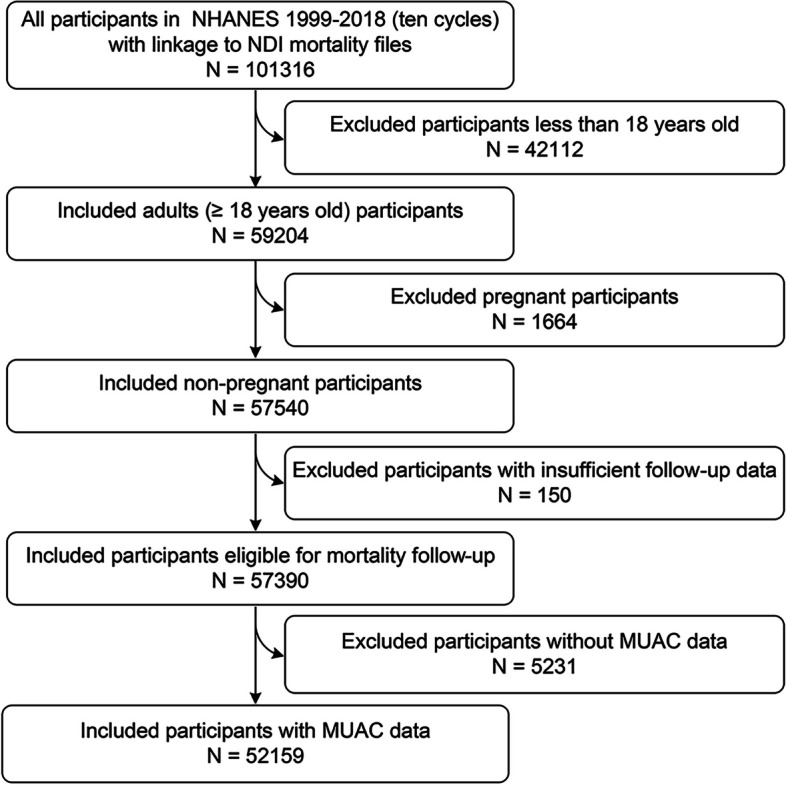
Flowchart of the present study indicating the included and excluded participants

A total of 101,316 participants were enrolled in ten cycles of NHANES from 1999 to 2018, and in our cohort study, 42,112 participants with ages less than 18, 1664 pregnant participants, 150 participants with insufficient follow-up information, and 5213 participants without MUAC data were successively excluded. The remaining 52,159 eligible participants were ultimately included in our main analyses (Fig. 1).

### Measurement of MUAC

The MUAC was measured with the participant standing upright with the arms hanging loosely without flexing or tightening the arm muscles. The examiner stood facing the participant’s right side and wrapped the measuring tape around the arm snugly at the midpoint level of the upper arm without compressing the skin. The tape was positioned perpendicular to the upper arm's long axis with two ends overlapping and the result lying on the lateral aspect of the arm. The midpoint of the upper arm refers to the posterior midpoint between the uppermost edge of the posterior border of the acromion process and the tip of the olecranon process when the right arm is bent 90° at the elbow. The MUAC was recorded to the nearest millimeter [15].

### Assessment of mortality

Mortality status for NHANES participants was ascertained through probabilistic record matching with the NDI Public-Use Linked Mortality Files through December 31, 2019 [16]. The primary outcome of this study was all-cause mortality. The secondary outcome was cause-specific mortality, defined according to the International Classification of Diseases, 10th Revision (ICD-10) codes recorded as the underlying leading cause of death: cardiovascular disease (CVD) mortality corresponding to codes I00-I09, I11, I13, I20-I51, and I60-I69; cancer mortality corresponding to codes C00-C97; and respiratory mortality corresponding to J09-J18 and J40-J47.

For the 1999–2018 NHANES, follow-up time was calculated using person months from examination to death or the end of the follow-up period, December 31, 2019, whichever came first.

### Assessment of covariates

The demographic characteristics were collected by standardized questionnaires during the household interview. Race/ethnicity was categorized into non-Hispanic White (white), non-Hispanic Black (black), Mexican American (Mexican), and other. Educational attainment was classified as below high school, high school or equivalent, and college or above. Marital status was grouped into never married, married, and other. The family poverty-to-income ratio (PIR) evaluates the economic level, which is a ratio of family income to the poverty threshold, ranging from 0 to 5, and a higher value indicates higher family income per capita.

The physical examinations were performed at the Mobile Examination Center, and the body mass index (BMI) was calculated as the weight in kilograms divided by the square of height in meters. Smoking status was determined based on whether participants were never smokers (smoked < 100 cigarettes/lifetime), former smokers (≥ 100 cigarettes/lifetime but do not currently smoke), or current smokers. Alcohol users were classified into never (had < 12 drinks in a lifetime), former (had ≥ 12 drinks in a lifetime but did not drink last year), mild (≤ 1 drink per day for women or ≤ 2 drinks per day for men), moderate (≤ 2 drinks per day for women or ≤ 3 drinks per day for men), and heavy (> two drinks per day for women or > three drinks per day for men), in which a drink referred to the alcoholic drink-equivalent of 12 oz of beer, 4 oz of wine, or 1 oz of liquor (such as whiskey or gin) [17]. Caffeine consumption was evaluated as the mean consumption from 2 typical days at 3- to 10-day intervals and calculated in mg/day. Diet quality was assessed by the Healthy Eating Index-2015 (HEI-2015), which measures adherence to the 2015–2020 Dietary Guidelines for Americans, and a higher score indicates a healthier diet [18]. Physical activity was measured by the NHANES Physical Activity Questionnaire and quantified in metabolic equivalent tasks (METs) multiplied by exercise time (minutes) per week (METs-min/week) [19].

Comorbid conditions were evaluated by combined information from NHANES datasets as our previous study, including hypertension, diabetes, coronary heart disease (CHD), chronic obstructive pulmonary disease (COPD), stroke, cancer, and chronic kidney disease (CKD) [20].

### Statistical analysis

Sample weights, pseudo-PSU (sdmvpsu), and pseudostratum (sdmvstra) were used in the data analyses to account for the stratified, multistage probability design of NHANES in which the combined sample weight was calculated as 2/5*WTMEC4YR for 1999–2002 and 1/5*WTMEC2YR for other survey cycles according to the NHANES analytic guidelines [21, 22].

Data are presented as the survey-weighted mean (standard error, SE) for continuous variables or number (survey-weighted percentage, %) for categorical variables. Survey-weighted ANOVA and the chi-square test were used to detect significant differences between the means and proportions between groups.

Survey-weighted Kaplan‒Meier survival analysis was used to compare the cumulative survival in different MUAC quartiles with the log-rank test. Survey-weighted Cox proportional hazards models were used to estimate the hazard ratios (HRs) and 95% confidential intervals (CIs) for the association of MUAC quartiles with all-cause and cause-specific mortality with the first quartile as the reference. According to the STROBE reporting guideline [14], the crude and multivariable models with different covariate adjustments were exhibited, in which sex, age, and race were adjusted in Model 1; education, marital status, and PIR were additionally adjusted in Model 2; and BMI, smoke, alcohol use, caffeine consumption, HEI-2015, physical activity, comorbidity or history of hypertension, diabetes, CHD, stroke, COPD, cancer, and CKD were additionally adjusted in Model 3. Moreover, MUACs were additionally calculated as a continuous variable to estimate the mortality risk change with each centimeter increment in MUAC in different multivariate models. In the primary analyses, no assumptions were used for missing data, and the listwise deletion method was used for missing variables in multivariate models.

To evaluate the potential nonlinear association of mortality with MUAC, restricted cubic spline (RCS) analysis was performed in multivariable-adjusted models, in which the number of knots was determined according to the minor Akaike information criterion (AIC) values.

The subgroup analyses were performed according to the dichotomic stratified variables, namely, sex (female or male), age at recruitment (≤ 60 vs. > 60), race (white vs. nonwhite), education level (high school or below vs. college or above), marital status (married vs. other), PIR (≤ 1.3 vs. > 1.3), BMI (≤ 28 vs. > 28), smoker (smoker vs. nonsmoker), drinker (current drinker vs. not), caffeine consumption (less than the median vs. more than the median), HEI-2015 (< 50 vs. ≥ 50), physical activity (physically active [≥ 600 MET-min/week] vs. physically inactive [< 600 MET-min/week]), and with vs. without hypertension, diabetes, stroke, COPD, cancer, and CKD, using the fully adjusted model except for the specific stratification variable. The likelihood ratio test also inspected interactions of MUAC with stratification variables.

Several sensitivity analyses were performed to test the robustness of the results. First, the primary analyses were repeated after excluding participants with extremely low or high MUACs (less than 1.5*interquartile range (IQR) below Q1 or more than 1.5*IQR above Q3) to test the effect of potential outliers. Second, participants with hypoalbuminemia (serum albumin < 35 g/L) were excluded to evaluate the impact of malnutrition on the relationship between MUAC and mortality. Third, we excluded events within the first two years of follow-up to reduce potential reverse causation. Fourth, we imputed all missing covariates using Fully Conditional Specification (FCS) implemented by the MICE algorithm [23] to test the influence of these missing variables.

Data were analyzed using the statistical packages in the R program (The R Foundation; http://www.r-project.org; version 4.2.1) and EmpowerStats (www.empowerstats.net, X&Y solutions, Inc. Boston, Massachusetts; version 4.1). A two-sided *P* < 0.05 was considered statistically significant.

## Results

### Baseline characteristics of participants

Among the 52,159 participants, representing 212 million US adults, 26,147 (50.9%) were female; the mean (SE) age was 46.1 (0.2) years; 22,368 (68.1%) were non-Hispanic white. The median MUAC of the participants was 32.5 (cm; IQR, 29.3–36.0); according to the quartiles, the MUAC values (cm) were categorized into four groups: Q1 (≤ 29.3), Q2 (29.4, 32.5), Q3 (32.6, 36.0), and Q4 (≥ 36.1).

During a median follow-up time of 112 (IQR, 60–170) months, a total of 7517 deaths were documented, of which 1934 (24.6%) were attributed to diseases of the heart, 1713 (24.0%) were attributed to malignant neoplasms, 404 (4.9%) were attributed to cerebrovascular diseases, 402 (6.2%) were attributed to chronic lower respiratory diseases, and 154 (1.8%) were attributed to influenza and pneumonia; the leading causes of death were reorganized into cardiovascular disease, cancer, and respiratory disease in the following analyses (Figure S1).

The population baseline characteristic profiles were compared across the quartiles of MUAC (Table 1). Briefly, a higher proportion of female, white, and nonmarried individuals and individuals with higher education levels tended to have a small MUAC; furthermore, individuals with a small MUAC tended to have lower BMI, lower PIR, lower caffeine consumption, less physical activity, higher HEI-2015 and a higher prevalence of stroke, cancer, COPD, and CKD but lower prevalence of diabetes, CHD, and hypertension (Table 1).

**Table 1:** Baseline characteristics of participants in different MUAC quartiles

	**Total**	**Q1 (≤ 29.3)**	**Q2 (29.4, 32.5)**	**Q3 (32.6, 36.0)**	**Q4 (≥ 36.1)**	***P*-value**
Number of participants (n)	52159	13062	13325	13019	12753	
Sex, n (%)						< 0.001
Female	26174(50.9)	8885(74.4)	6314(50.5)	5263(38.7)	5712(41.6)	
Male	25985(49.1)	4177(25.6)	7011(49.5)	7756(61.3)	7041(58.4)	
Age (years)	46.1(0.2)	45.4(0.3)	47.3(0.3)	46.8(0.2)	45.0(0.2)	< 0.001
Race, n (%)						< 0.001
White	22368(68.1)	5846(69.2)	5738(68.2)	5550(68.4)	5234(66.6)	
Black	11214(11.1)	2114(8.1)	2460(9.7)	2736(10.6)	3904(15.9)	
Mexican	9534(8.3)	2121(6.7)	2683(8.9)	2705(9.5)	2025(8.0)	
Other	9043(12.5)	2981(16.1)	2444(13.2)	2028(11.5)	1590(9.6)	
Education, n (%)						< 0.001
Below high school	13057(16.6)	3019(17.1)	3687(19.1)	3483(17.5)	2868(15.3)	
High school or equivalent	11254(23.3)	2480(21.8)	2824(23.8)	2863(24.3)	3087(26.4)	
College or above	24076(56.6)	5923(61.1)	5890(57.1)	6060(58.2)	6203(58.2)	
Marital status, n (%)						< 0.001
Never married	10318(18.3)	3107(22.8)	2471(18.1)	2245(16.5)	2495(18.4)	
Married	25318(53.7)	5454(49.7)	6697(55.7)	6749(58.5)	6418(57.3)	
other	14395(24.9)	3701(27.5)	3636(26.2)	3614(24.9)	3444(24.4)	
PIR	3.0(0.0)	2.9(0.0)	3.0(0.0)	3.1(0.0)	3.0(0.0)	< 0.001
BMI (kg/m^2)^	28.6(0.1)	22.0(0.0)	26.0(0.0)	29.4(0.0)	36.5(0.1)	< 0.001
Smoker, n (%)						< 0.001
Never	26830(52.5)	6764(56.2)	6778(53.3)	6577(52.0)	6711(54.0)	
Former	12003(23.9)	2340(19.9)	3124(24.3)	3360(26.8)	3179(26.8)	
Now	10395(21.1)	2686(23.9)	2688(22.4)	2607(21.2)	2414(19.2)	
Alcohol user, n (%)						< 0.001
Never	6713(10.5)	1965(14.1)	1744(12.2)	1518(10.3)	1486(10.1)	
Former	7761(12.8)	1709(12.5)	1973(13.6)	1951(14.1)	2128(16.4)	
Mild	14868(32.2)	3350(33.7)	3960(37.1)	3920(36.9)	3638(34.8)	
Moderate	6683(15.3)	1721(20.0)	1632(16.4)	1715(16.2)	1615(15.6)	
Heavy	9063(19.4)	1903(19.7)	2253(20.7)	2419(22.4)	2488(23.1)	
Caffeine consumption (mg/day)	175.1(2.2)	156.5(3.4)	175.3(3.3)	186.0(3.4)	180.9(3.2)	< 0.001
Physical activity (METs-min/week)	3418.6(59.5)	2892.0(70.1)	3397.3(91.8)	3568.2(79.7)	3782.9(93.5)	< 0.001
HEI-2015	50.2(0.2)	51.8(0.3)	51.3(0.2)	49.8(0.2)	47.9(0.2)	< 0.001
Hypertension, n (%)						< 0.001
No	30872(63.2)	8889(72.6)	8313(67.6)	7498(61.9)	6172(51.6)	
Yes	21287(36.8)	4173(27.4)	5012(32.4)	5521(38.1)	6581(48.4)	
Diabetes, n (%)						< 0.001
No	43662(87.6)	11865(93.9)	11417(90.0)	10747(87.3)	9633(79.9)	
Yes	8493(12.3)	1196(6.1)	1908(10.0)	2269(12.7)	3120(20.1)	
CHD, n (%)						0.010
No	46203(93.0)	10917(97.0)	11841(96.8)	11827(96.1)	11618(96.3)	
Yes	2029(3.3)	470(3.0)	519(3.2)	531(3.9)	509(3.7)	
Stroke, n (%)						
No	46615(93.9)	10972(97.1)	11959(97.5)	11964(97.5)	11720(97.3)	
Yes	1770(2.6)	462(2.9)	441(2.5)	432(2.5)	435(2.7)	
COPD, n (%)						0.040
No	46469(92.9)	10908(95.5)	11924(96.3)	11955(96.3)	11682(96.2)	
Yes	2016(3.8)	555(4.5)	502(3.7)	464(3.7)	495(3.8)	
Cancer, n (%)						< 0.001
No	43865(87.3)	10221(89.1)	11140(89.5)	11341(91.4)	11163(91.7)	
Yes	4535(9.2)	1220(10.9)	1267(10.5)	1060(8.6)	988(8.3)	
CKD, n (%)						< 0.001
No	40327(81.6)	9701(83.7)	10451(86.4)	10357(87.4)	9818(84.7)	
Yes	9061(13.8)	2497(16.3)	2218(13.6)	2040(12.6)	2306(15.3)	

Continuous variables are presented as survey-weighted mean (standard error) and categorical variables are presented as number (survey-weighted percentage, %)

*Abbreviations*: *BMI* body mass index, *PIR* family income-to-poverty ratio, *HEI* Healthy Eating Index, *COPD* chronic obstructive pulmonary disease, *CHD* coronary heart disease, *CKD* chronic kidney disease

### Associations of MUAC quartiles with all-cause and cause-specific mortality

**Fig. 2:**
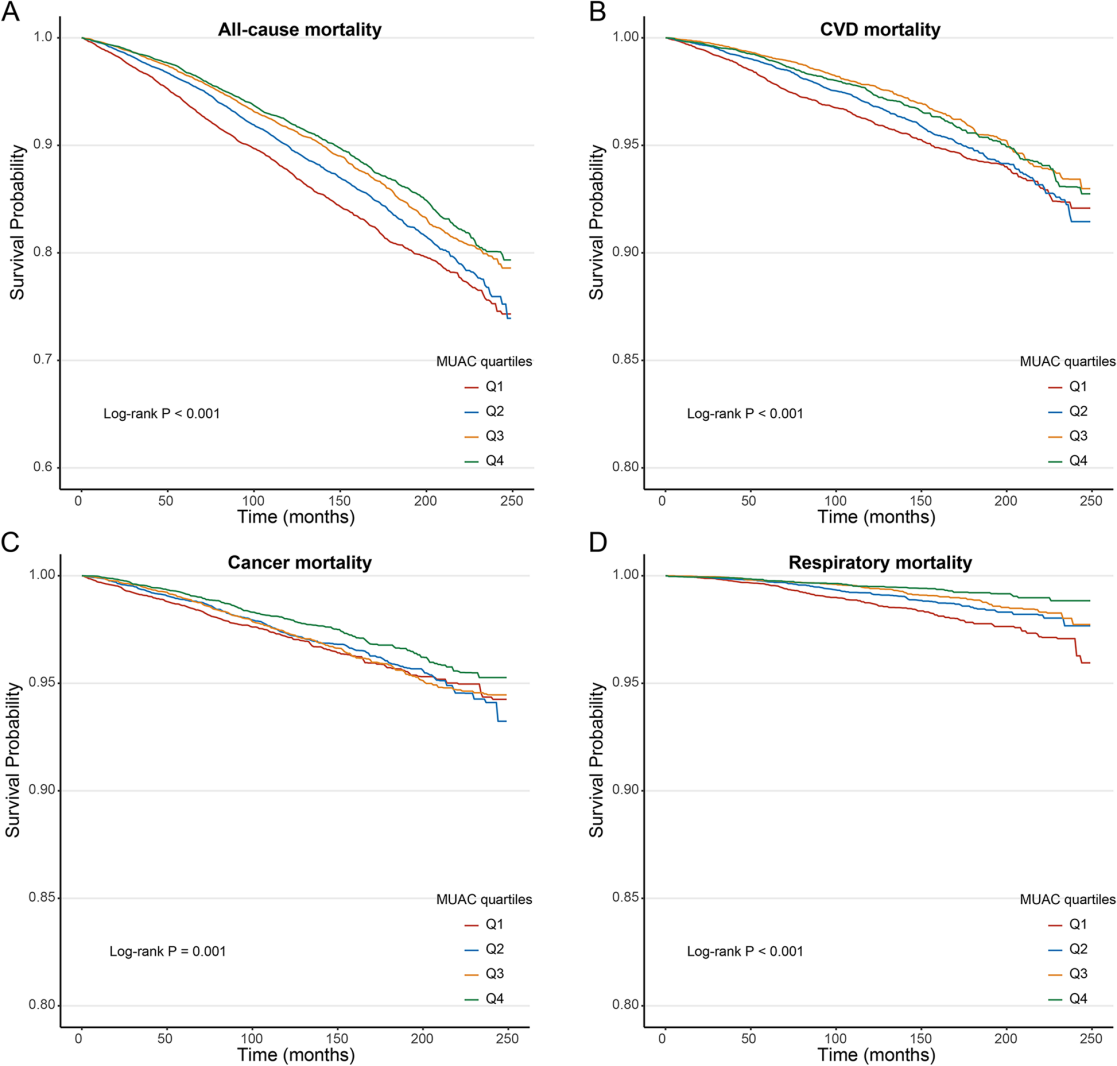
Survey-weighted Kaplan‒Meier survival curves and log-rank tests comparing mortality due to all-cause (**A**), CVD (**B**), cancer (**C**), and respiratory disease (**D**) in participants in different MUAC quartiles. CVD, cardiovascular disease

The unadjusted survey-weighted Kaplan‒Meier survival analyses, followed by the log-rank test, showed that MUAC was significantly inversely associated with all-cause and cause-specific mortality, in which individuals in the lowest quartiles of MUAC consistently had the highest mortality risk (Fig. 2).

After multivariable adjustment, the all-cause mortality risk was lower in the Q2-Q4 group of MUAC than in the Q1 group. Briefly, compared with the reference group (Q1), the HRs for all-cause mortality were estimated to be 0.76 (95% CI, 0.67–0.87) for the Q2 group, 0.69 (95% CI, 0.59–0.81) for the Q3 group, and 0.59 (95% CI, 0.46–0.75) for the Q4 group (Table 2). As a continuous linear variable, every centimeter increase increment in MUAC was associated with a 7% decrease reduction in all-cause mortality risk (HR: 0.93, 95%,0.91–0.95) (Table S2).

**Table 2:** Survey-weighted multivariate analyses of the associations of categorical MUAC or continuous linear MUAC with all-cause and cause-specific mortality

	**Q1 (≤ 29.3)**	**Q2 (29.4, 32.5)**		**Q3 (32.6, 36.0)**		**Q4 (≥ 36.1)**	
	**HR (95% CI)**	**HR (95% CI)**	***P*-value**	**HR (95% CI)**	***P*-value**	**HR (95% CI)**	***P*-value**
All-cause mortality							
Crude	1(ref)	0.84 (0.78, 0.91)	< 0.001	0.72 (0.66, 0.78)	< 0.001	0.67 (0.61, 0.73)	< 0.001
Model 1	1(ref)	0.72 (0.67, 0.77)	< 0.001	0.70 (0.64, 0.76)	< 0.001	0.80 (0.73, 0.88)	< 0.001
Model 2	1(ref)	0.73 (0.68, 0.79)	< 0.001	0.73 (0.67, 0.80)	< 0.001	0.82 (0.75, 0.90)	< 0.001
Model 3	1(ref)	0.76 (0.67, 0.87)	< 0.001	0.69 (0.59, 0.81)	< 0.001	0.59 (0.46, 0.75)	< 0.001
CVD mortality							
Crude	1(ref)	0.87 (0.77, 0.99)	0.029	0.68 (0.60, 0.77)	< 0.001	0.73 (0.63, 0.84)	< 0.001
Model 1	1(ref)	0.76 (0.67, 0.85)	< 0.001	0.72 (0.63, 0.82)	< 0.001	1.01 (0.88, 1.15)	0.932
Model 2	1(ref)	0.78 (0.68, 0.89)	< 0.001	0.75 (0.65, 0.88)	< 0.001	1.04 (0.91, 1.19)	0.564
Model 3	1(ref)	0.73 (0.58, 0.93)	0.010	0.57 (0.43, 0.74)	< 0.001	0.52 (0.35, 0.77)	0.001
Cancer mortality							
Crude	1(ref)	0.94 (0.79, 1.10)	0.428	0.94 (0.81, 1.10)	0.449	0.74 (0.63, 0.87)	< 0.001
Model 1	1(ref)	0.76 (0.64, 0.90)	0.002	0.83 (0.70, 0.98)	0.030	0.78 (0.65, 0.94)	0.010
Model 2	1(ref)	0.79 (0.66, 0.95)	0.011	0.86 (0.72, 1.03)	0.109	0.81 (0.67, 0.98)	0.027
Model 3	1(ref)	0.94 (0.71, 1.24)	0.651	0.98 (0.69, 1.39)	0.906	0.75 (0.46, 1.24)	0.267
Respiratory mortality							
Crude	1(ref)	0.66 (0.51, 0.86)	0.002	0.53 (0.41, 0.68)	< 0.001	0.37 (0.25, 0.56)	< 0.001
Model 1	1(ref)	0.60 (0.46, 0.79)	< 0.001	0.59 (0.45, 0.78)	< 0.001	0.54 (0.35, 0.82)	0.004
Model 2	1(ref)	0.58 (0.43, 0.37)	< 0.001	0.62 (0.46, 0.83)	0.001	0.52 (0.35, 0.77)	0.001
Model 3	1(ref)	0.57 (0.37, 0.87)	0.009	0.75 (0.46, 1.22)	0.247	0.39 (0.19, 0.83)	0.014

Data were calculated by svycoxph to fit a multivariate Cox proportional hazards model to data from a complex survey design

Model 1: Adjusted for sex, age, and race

Model 2: Adjusted for sex, age, race, education, marital status, and family income-to-poverty ratio

Model 3: Adjusted for sex, age, race, education, marital status, family income-to-poverty ratio, BMI, smoking, alcohol use, caffeine consumption, HEI-2015, physical activity, comorbidity or history of hypertension, diabetes, CHD, stroke, COPD, cancer, and CKD

*Abbreviations*: *HR* hazard ratio, *CI* confidential interval, *BMI* body mass index, *PIR* family income-to-poverty ratio, *HEI* Healthy Eating Index, *COPD* chronic obstructive pulmonary disease, *CHD* coronary heart disease, *CKD* chronic kidney disease

Similarly, CVD mortality was consistently lower in the Q2-Q3 group of MUAC than in the Q1 group in all models. After being fully adjusted, the HRs for all-cause mortality were estimated to be 0.73 (95% CI, 0.58–0.93) for the Q2 group, 0.57 (95% CI, 0.43–0.74) for the Q3 group, and 0.52 (95% CI, 0.35–0.77) for the Q4 group compared with the Q1 group (Table 2). As a continuous linear variable, every centimeter increase in MUAC was associated with a 6% decrease in CVD mortality risk (HR: 0.94, 95%, 0.89–0.98) (Table S2).

Moreover, respiratory mortality was consistently lower in the Q2 group of MUAC than in the Q1 group. With full adjustment, the HRs for respiratory mortality were estimated to be 0.57 (95% CI, 0.37–0.87) for the Q2 group, 0.75 (95% CI, 0.46–1.22) for the Q3 group, and 0.39 (95% CI, 0.19–0.83) for the Q4 group compared with the Q1 group (Table 2). As a continuous linear variable, every centimeter increase in MUAC was associated with an 11% decrease in respiratory mortality risk (HR: 0.89, 95% CI, 0.82–0.97) (Table S2).

However, no consistent differences in cancer mortality were observed between the Q2-Q4 and Q1 groups. With the full adjustment, the HRs for cancer mortality were estimated to be 0.94 (95% CI, 0.71–1.24) for the Q2 group, 0.98 (95% CI, 0.69–1.39) for the Q3 group, and 0.75 (95% CI, 0.46–1.24) for the Q4 group compared with the Q1 group (Table 2), although every centimeter increase in MUAC was associated with an 8% decrease in cancer mortality risk (HR: 0.92, 95% CI, 0.87–0.96) (Table S2).

### Nonlinear analyses of the association between continuous MUAC and mortality

**Fig. 3:**
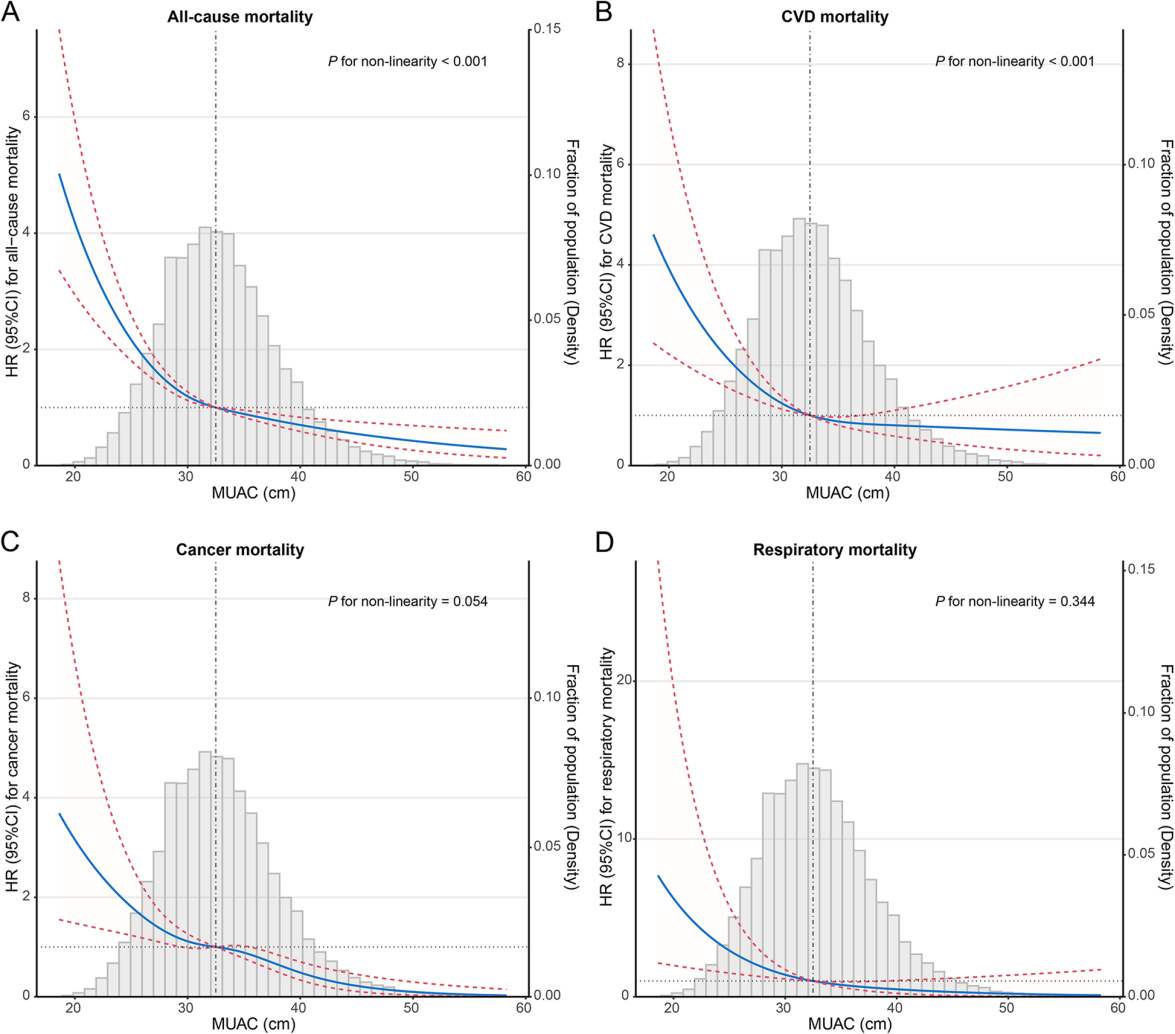
Survey-weighted restricted cubic spline analyses of the associations of continuous MUAC with all-cause mortality (**A**), CVD mortality (**B**), cancer mortality (**C**), and respiratory mortality (**D**), and the probability distribution histogram is represented in the background. The models were all adjusted for sex, age, race, education, marital status, family income-to-poverty ratio, BMI, smoking status, alcohol use, caffeine consumption, HEI-2015, physical activity, comorbidity or history of hypertension, diabetes, CHD, stroke, COPD, cancer, and CKD. Solid blue lines are multivariable-adjusted HR estimations, and the dashed red lines are the corresponding 95% CIs. The reference point was set at the median (32.5 cm)

Spline models with covariates fully adjusted were constructed to profile a more direct relationship between MUAC and mortality. An L-shaped association was observed between MUAC and all-cause mortality (nonlinear *P* < 0.001) in which all-cause mortality risk decreased steeply until approximately the median MUAC, and then it plateaued (Fig. 3A). Similarly, a more apparent L-shaped association was observed between MUAC and CVD mortality (nonlinear *P* < 0.001, Fig. 3B). However, no significant nonlinear relationship was detected between MUAC and cancer and respiratory mortality (nonlinear *P* > 0.05, Fig. 3C, D).

### Subgroup analyses of the association of MUAC with mortality

Subgroup analyses were performed to depict the different potential associations of MUAC quartiles and mortality in subpopulations. Significant interactions were found between MUAC and sex in which the inverse association of MUAC with all-cause mortality was observed only in men and was not significant in women (Table 3). Additionally, in elderly individuals (> 60 years), the detrimental association of the low MUAC quartile with all-cause mortality was more prominent, which was confirmed by the interaction test indicating the additional modification effect of categorical age (Table 3). Similarly, BMI was another major modification factor. The inverse association of MUAC with all-cause mortality was observed only in individuals with BMI ≤ 28 but not in those with high BMI (Table 3). Other association differentials are also listed in Table 3. Similarly, subgroup analyses were also performed on the association of MUAC quartiles and CVD mortality (Table S3), cancer mortality (Table S4), and respiratory mortality (Table S5). The differences in population distribution in MUAC quartiles between subgroups could partially explain the discrepancy of size effect of MUAC in association with mortality risk in different subgroups (Table S6).

**Table 3:** Subgroup analyses of the associations of MUAC quartiles with all-cause mortality and interaction analyses of MUAC with stratification variables

Subgroup	No. of participants	Q1 (≤ 29.3)	Q2 (29.4, 32.5)	Q3 (32.6, 36.0)	Q4 (≥ 36.1)	P for trend	P for interaction
Sex							0.024
Female	26174	1(ref)	0.98(0.83,1.16)	0.92(0.71,1.17)	0.82(0.55,1.24)	0.359	
Male	25985	1(ref)	0.61(0.51,0.75)	0.54(0.44,0.67)	0.43(0.31,0.60)	< 0.001	
Age							< 0.001
≤ 60	36632	1(ref)	0.78(0.58,1.03)	0.69(0.53,0.92)	0.55(0.35,0.85)	0.007	
> 60	15527	1(ref)	0.69(0.60,0.80)	0.56(0.45,0.68)	0.39(0.28,0.54)	< 0.001	
Race							0.022
White	22368	1(ref)	0.73(0.62,0.85)	0.66(0.55,0.80)	0.55(0.41,0.74)	< 0.001	
Non-white	29791	1(ref)	1.01(0.77,1.34)	0.90(0.66,1.21)	0.82(0.54,1.23)	0.267	
Education							0.680
High school or below	24311	1(ref)	0.72(0.60,0.86)	0.68(0.55,0.85)	0.56(0.40,0.80)	0.002	
College or above	24076	1(ref)	0.81(0.68,0.96)	0.71(0.57,0.88)	0.61(0.45,0.81)	< 0.001	
Marital status							0.582
Married	25318	1(ref)	0.75(0.64,0.89)	0.65(0.52,0.82)	0.56(0.40,0.79)	< 0.001	
other	24713	1(ref)	0.77(0.63,0.93)	0.73(0.59,0.90)	0.60(0.43,0.83)	0.001	
PIR							0.113
≤ 1.3	15218	1(ref)	0.95(0.75,1.21)	1.02(0.75,1.40)	1.11(0.71,1.76)	0.636	
> 1.3	32346	1(ref)	0.72(0.62,0.83)	0.61(0.51,0.72)	0.47(0.36,0.63)	< 0.001	
BMI							0.011
≤ 28	27361	1(ref)	0.78(0.68,0.89)	0.66(0.55,0.80)	0.68(0.28,1.64)	< 0.001	
> 28	24472	1(ref)	1.71(1.02,2.86)	1.48(0.93,2.34)	1.29(0.79,2.09)	0.014	
Smoker							0.336
Non-smoker	26830	1(ref)	0.75(0.62,0.92)	0.71(0.56,0.90)	0.52(0.35,0.76)	< 0.001	
Smoker	22398	1(ref)	0.74(0.63,0.87)	0.66(0.53,0.83)	0.61(0.44,0.86)	0.005	
Current drinker							0.721
No	14474	1(ref)	0.84(0.68,1.02)	0.81(0.62,1.04)	0.72(0.51,1.02)	0.063	
Yes	30614	1(ref)	0.72(0.60,0.86)	0.61(0.50,0.75)	0.50(0.36,0.71)	< 0.001	
Caffeine consumption							0.218
≤ P50	22398	1(ref)	0.84(0.68,1.04)	0.81(0.64,1.01)	0.59(0.41,0.83)	0.004	
> P50	22341	1(ref)	0.71(0.60,0.85)	0.62(0.50,0.78)	0.57(0.42,0.79)	< 0.001	
HEI-2015							0.254
≤ 50	25240	1(ref)	0.76(0.61,0.95)	0.72(0.57,0.90)	0.67(0.46,0.99)	0.045	
> 50	24233	1(ref)	0.77(0.65,0.92)	0.68(0.54,0.84)	0.51(0.36,0.72)	< 0.001	
Physical activity							0.452
Inactive	13509	1(ref)	0.82(0.69,0.98)	0.72(0.57,0.90)	0.68(0.49,0.95)	0.014	
Active	24356	1(ref)	0.71(0.59,0.85)	0.66(0.52,0.83)	0.50(0.35,0.70)	< 0.001	
Hypertension							0.123
No	30872	1(ref)	0.77(0.62,0.97)	0.77(0.56,1.04)	0.82(0.52,1.30)	0.387	
Yes	21287	1(ref)	0.75(0.64,0.87)	0.64(0.53,0.78)	0.48(0.35,0.64)	< 0.001	
Diabetes							0.260
No	43662	1(ref)	0.75(0.65,0.87)	0.72(0.60,0.87)	0.64(0.48,0.86)	0.003	
Yes	8493	1(ref)	0.84(0.63,1.13)	0.64(0.47,0.89)	0.48(0.32,0.71)	< 0.001	
CHD							0.650
No	46203	1(ref)	0.77(0.67,0.88)	0.72(0.61,0.85)	0.61(0.47,0.80)	< 0.001	
Yes	2029	1(ref)	0.76(0.53,1.09)	0.53(0.34,0.84)	0.43(0.24,0.79)	0.004	
Stroke							0.265
No	46615	1(ref)	0.75(0.65,0.86)	0.69(0.58,0.81)	0.58(0.45,0.74)	< 0.001	
Yes	1770	1(ref)	0.82(0.53,1.27)	0.60(0.35,1.02)	0.55(0.25,1.19)	0.090	
COPD							0.514
No	46469	1(ref)	0.75(0.66,0.86)	0.66(0.57,0.77)	0.56(0.44,0.72)	< 0.001	
Yes	2016	1(ref)	0.81(0.52,1.26)	0.97(0.52,1.80)	0.67(0.27,1.64)	0.520	
Cancer							0.482
No	43865	1(ref)	0.80(0.68,0.94)	0.70(0.58,0.85)	0.58(0.44,0.76)	< 0.001	
Yes	4535	1(ref)	0.69(0.55,0.87)	0.68(0.49,0.94)	0.68(0.44,1.04)	0.071	
CKD							0.570
No	40327	1(ref)	0.79(0.66,0.94)	0.72(0.59,0.88)	0.62(0.45,0.86)	0.004	
Yes	9061	1(ref)	0.74(0.60,0.91)	0.65(0.51,0.83)	0.54(0.37,0.78)	< 0.001	

Test for trend was based on the variable containing the median value for each quartile

*Abbreviations*: *CI* confidence interval, *BMI* body mass index, *PIR* family income-to-poverty ratio, *P50* 50th percentile, *HEI* Healthy Eating Index, *COPD* chronic obstructive pulmonary disease, *CHD* coronary heart disease, *CKD* chronic kidney disease

Due to the interaction effects detected between subgroup variables and MUAC, the linear association of continuous MUAC with all-cause and cause-specific mortality stratified by subgroup variables was thoroughly assessed in Table S7, in which mortality risk corresponding to each centimeter change of MUAC varied significantly in subgroup of sex, age, PIR, and BMI (Table S7). For potential differentials in nonlinear associations of MUAC with mortality in different subgroups with major interaction effects, RCS analyses were performed in participants of females and males, ages > 60 and ≤ 60, with BMI > 28 and ≤ 28, respectively. In men, with the increase in MUAC, the all-cause mortality and cause-specific mortality decreased dramatically when not reaching the median (Figure S2). Similar associations with all-cause mortality and CVD mortality were also found in individuals > 60 years (Figure S3). Similar associations with all-cause mortality were found in individuals with BMI ≤ 28 (Figure S4).

### Sensitivity analyses of the association of MUAC with mortality

The association of MUAC with all-cause mortality and cause-specific mortality was robust in all sensitivity analyses. Briefly, after excluding participants with potential MUAC outliers (*n* = 903), the association of categorical MUAC or continuous linear with all-cause mortality and cause-specific mortality did not change materially (Table S8). Moreover, similar analysis results were also observed after excluding participants with possible hypoalbuminemia (*n* = 3480, including 600 serum albumin less than 35 g/L and 2880 missing serum albumin data) (Table S9) or excluding participants who died in the first two years (*n* = 953) (Table S10). In addition, the imputation of all missing covariates also did not cause substantial shifts in the results (Table S11)*.*

## Discussion

**Fig. 4:**
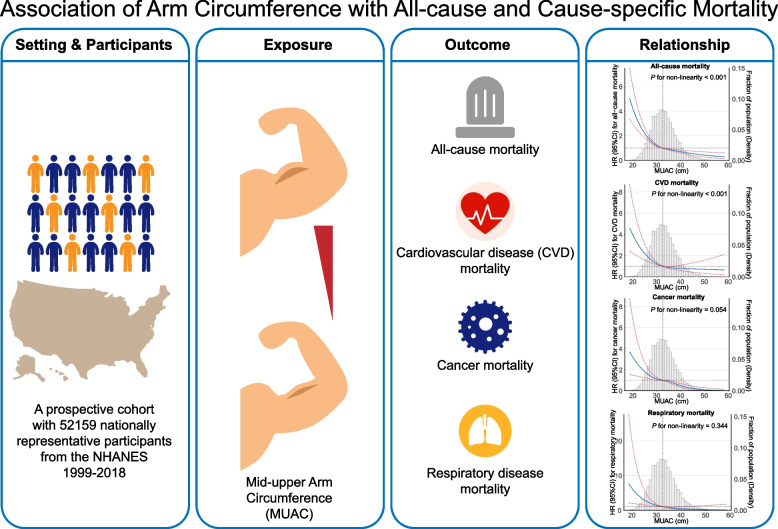
The L-shaped association of mid-upper arm circumference with all-cause and cause-specific mortality in the US. The nationally representative cohort from the National Health and Nutrition Examination Survey (1999—2018) was analyzed with mortality information obtained through linkage to the National Death Index. MUAC was non-linearly associated with all-cause mortality and CVD mortality in an L-shaped pattern

In this nationally representative cohort of US adults, inverse associations were detected between MUAC and all-cause mortality, CVD mortality, cancer mortality, and respiratory mortality. Adults with MUAC in the first quartile tended to have higher risks of all-cause mortality, CVD mortality, and respiratory mortality. Furthermore, L-shaped nonlinear dose–response associations of MUAC with all-cause mortality and CVD mortality were observed. Moreover, sex, age, and BMI may have additional modification effects on the association of MUAC with all-cause mortality and cause-specific mortality, in which more prominent inverse associations were observed in male, elderly, and non-overweight individuals (Fig. 4).

The WHO has recommended MUAC as an essential tool to assess the status of nutrition and growth in children 6–60 months of age [24, 25], but its application in adults has yet to be universally acknowledged [26]. According to the original measurement data we collected, the median MUAC of our participants was 32.5 cm, which was similar to a previous investigation [27] but higher than that from a Chinese cohort [28] and a Bangladesh cohort [29], which was possibly caused by differences in population composition, economic development, and general lifestyle; thus, it should be reasonable to set the reference value according to the specific or local population in public health evaluation.

We validated the inverse association of MUAC with all-cause mortality, in which individuals in lower MUAC quartiles tended to have higher mortality risk. The dose–response relation was more prominent when the MUAC was less than the median value. This was consistent with previous population-based studies, including another American cohort [13], a Canadian cohort [30], a European cohort [31], and two Asian cohorts [28, 29]. However, another recent study did not detect a significant association between arm circumference and mortality with NHANES datasets [12], possibly due to the differences in sample size and inclusion criteria, as the arm circumference values in their results were more significant than ours.

For the cause of death, we observed that among the top three causes, MUAC was inversely associated with CVD mortality and respiratory mortality. The Bangladesh cohort also reported the inverse association of MUAC with CVD mortality [29]. Nevertheless, it was insignificant in another study from NHANES datasets [13], possibly due to differences in sample size, categorization of MUAC, and strategy of covariate adjustment. The respiratory prognostic role of MUAC in adults has rarely been reported. Due to the limited sample size of respiratory mortality, more studies are still needed to confirm the association. The association of MUAC with cancer mortality was not independent, similar to other research [13, 29, 32, 33], possibly due to the heterogeneity of cancer and the unpredictability of the incidence of malignancy from basic anthropometric measurements.

The more prominent associations of MUAC with mortality in men, elderly individuals, and non-overweight individuals implied a more valuable role of MUAC in health evaluation in these populations. The inverse association of arm circumference with mortality in men was also reported by a 50-year follow-up study from an Italian cohort [34] and another American cohort [35]. Similarly, the higher risk of mortality for elderly individuals with a small arm circumference was consistent with previous studies focused on the elderly population [6–8, 36]. The poor prognosis indicator of small arm circumference in thin adults may be related to malnutrition or overall hypofunction of the musculoskeletal system, as reported by others [13, 29]. Taken together, males, elderly individuals, and non-overweight individuals would benefit more from a large arm circumference, and vice versa, small arm circumferences may deserve more attention in health evaluations for these individuals.

Compared with commonly used anthropometric indicators for adults, arm circumference may have some unique roles in health evaluation. Similarly to our results, a Chinese adult cohort study also showed that the association of MUAC with all-cause mortality was independent of BMI [28]. Moreover, in elderly individuals, MUAC loss is more strongly and consistently associated with increased mortality compared to BMI loss [6, 8]. Concerning other body circumference indicators, extremity circumferences, represented by arm, calf, or thigh circumference, showed opposite and independent effects on mortality risk to central circumference, represented by waist circumference, in which extremity circumferences are negatively associated with mortality risk whereas central circumference is positively associated with mortality risk [30, 37].

A detailed mechanism that could associate arm circumference with long-term mortality has yet to be fully recognized. First, the small arm circumference may reflect potential muscle impairment caused by physical inactivity, nutritional depletion, and systemic inflammation characterized in some chronic pathologic statuses [38]. Second, the dimension of the upper extremity may reflect the disturbance of systematic glucose metabolism, e.g., insulin resistance [39, 40]. Third, peripheral subcutaneous adipose tissue in the extremities could exhibit cardiovascular protective effects [41–43]. Arm circumference may indicate overall functional status but may be not the direct causal factor.

There are some strengths in our study. First, in a relatively large (> 50,000) and nationally representative cohort, we validated the L-shaped association of arm circumference with all-cause mortality and cause-specific mortality. Second, comprehensive analyses, including survey-weighted statistical strategy, adjustment of extensive objective covariates, and treating arm circumference as categorical and continuous variables with linear and nonlinear methods, evaluated the association adequately. Third, a series of sensitivity analyses guaranteed the robustness of our results.

However, we must acknowledge some limitations of our study. First, the association was found in the US adult population and still needs to be confirmed in other populations; meanwhile, the reference value for MUAC should be redefined according to the local people for public health practitioners. Second, the arm circumference in our study was measured once at baseline, but the dynamic change in arm circumference may be more meaningful in evaluating individual health and public health, which needs future studies to confirm. Third, the causal factors that lead to low arm circumference and high mortality risk have yet to be fully explored. In our future study, we will identify these factors as targets for public health intervention. Fourthly, the relatively extensive adjustment of confounders in our final model may lead to overfitting, which needs to be noticed in interpreting the results. Finally, the independent role of arm circumference in health evaluation or mortality risk prediction still needs further confirmation because residual or unmeasured confounding can only be partially excluded despite every effort to adjust for common confounding factors.

## Conclusions

In adults in the general US population, arm circumference was inversely associated with the risk of all-cause mortality, CVD mortality, and respiratory mortality. When the arm circumference was lower than the median value, the risk of all-cause mortality and CVD mortality increased dramatically with the decrease in arm circumference. The detrimental association of the low MUAC with mortality risk was more prominent in populations of male, elderly, and non-overweight individuals. Our results implied the valuable role of arm circumference in mortality risk prediction and public health evaluation.

### Supplementary Information

**Additional file 1: Figure S1.** The leading causes of death distribution in the enrolled participants with survey-weighted percentages.

**Additional file 2: Figure S2.** Survey-weighted restricted cubic spline analyses of the associations of continuous MUAC with all-cause mortality (A), CVD mortality (B), cancer mortality (C), and respiratory mortality (D) in male and female participants. Solid blue and red lines are multivariable-adjusted HR estimations, and the shaded areas are the corresponding 95% CIs. The reference points were set at the median of each subgroup.

**Additional file 3: Figure S3.** Survey-weighted restricted cubic spline analyses of the associations of continuous MUAC with all-cause mortality (A), CVD mortality (B), cancer mortality (C), and respiratory mortality (D) in participants with age > 60 and ≤ 60. Solid blue and red lines are multivariable-adjusted HR estimations, and the shaded areas are the corresponding 95% CIs. The reference points were set at the median of each subgroup.

**Additional file 4:** **Figure S4**. Survey-weighted restricted cubic spline analyses of the associations of continuous MUAC with all-cause mortality (A), CVD mortality (B), cancer mortality (C), and respiratory mortality (D) in participants with BMI > 28 and ≤ 28. Solid blue and red lines are multivariable-adjusted HR estimations, and the shaded areas are the corresponding 95% CIs. The reference points were set at the median of each subgroup.

**Additional file 5: Table S1. **STROBE checklist (v4) for cohort studies.

**Additional file 6: Table S2. **Survey-weighted multivariate analyses of the associations of continuous linear MUAC with all-cause and cause-specific mortality.

**Additional file 7: Table S3. **Subgroup analyses of the associations of MUAC quartiles with CVD mortality and interaction analyses of MUAC with stratification variables.

**Additional file 8: Table S4. **Subgroup analyses of the associations of MUAC quartiles with cancer mortality and interaction analyses of MUAC with stratification variables.

**Additional file 9: Table S5. **Subgroup analyses of the associations of MUAC quartiles with respiratory mortality and interaction analyses of MUAC with stratification variables.

**Additional file 10: Table S6.** Number and proportion (percentage) of participants in MUAC quartiles stratified by subgroup analysis variables.

**Additional file 11: Table S7.** Survey-weighted multivariate analyses of the associations of continuous linear MUAC with all-cause and cause-specific mortality in different subgroups.

**Additional file 12: Table S8.** Survey-weighted multivariate analyses of the associations of MUAC quartiles with all-cause and disease-specific mortality after excluding participants with potential MUAC outliers. (*n* = 903).

**Additional file 13: Table S9.** Survey-weighted multivariate analyses of the associations of MUAC quartiles with all-cause and disease-specific mortality after excluding participants with possible hypoalbuminemia . (*n* = 3480).

**Additional file 14: Table S10.** Survey-weighted multivariate analyses of the associations of MUAC quartiles with all-cause and disease-specific mortality after excluding participants who died in the first two years. (*n* = 953).

**Additional file 15: Table S11.** Survey-weighted multivariate analyses of the associations of MUAC quartiles with all-cause and disease-specific mortality after imputation of all missing covariates.

## Data Availability

The datasets analyzed in the current study are publicly available at the NHANES website: https://wwwn.cdc.gov/nchs/nhanes/Default.aspx.
