# Supplementary figures and images for: The L-shaped association of mid-upper arm circumference with all-cause and cause-specific mortality in US adults: a population-based prospective cohort study
Source: BMC Public Health. 2023 Nov 20;23:2297. doi: 10.1186/s12889-023-17064-x (PMC10662296; doi:10.1186/s12889-023-17064-x)

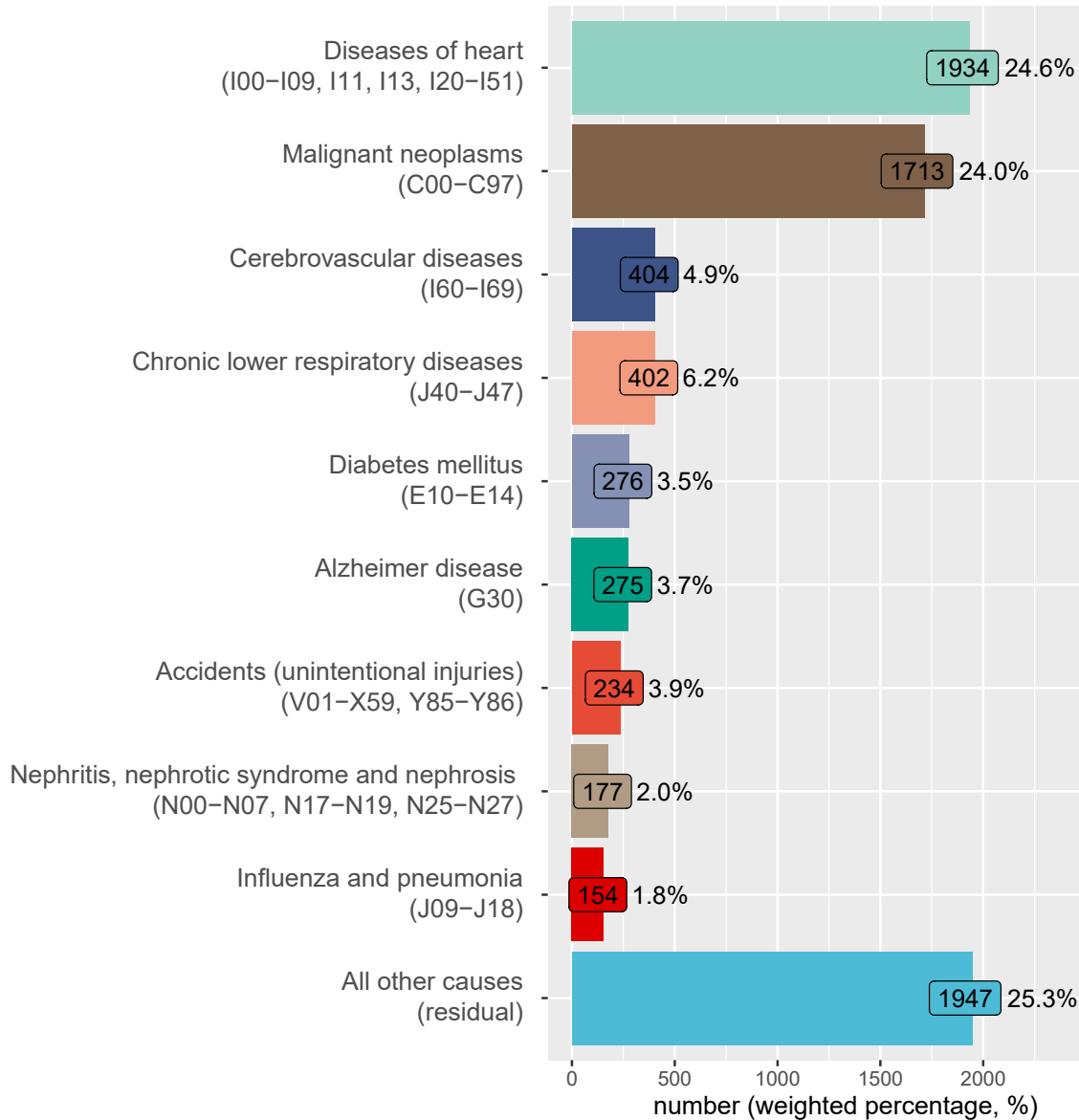

Supplement: Supplementary file 1 — Additional file 1: Figure S1. The leading causes of death distribution in the enrolled participants with survey-weighted percentages. [file 12889_2023_17064_MOESM1_ESM.pdf]

**A**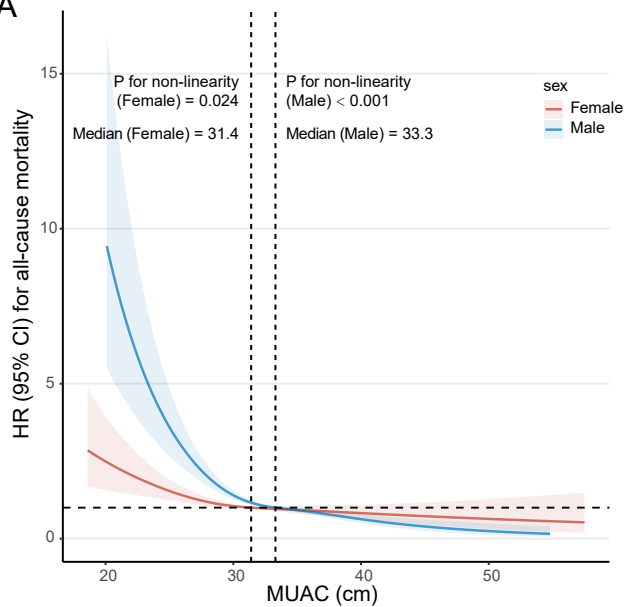**B**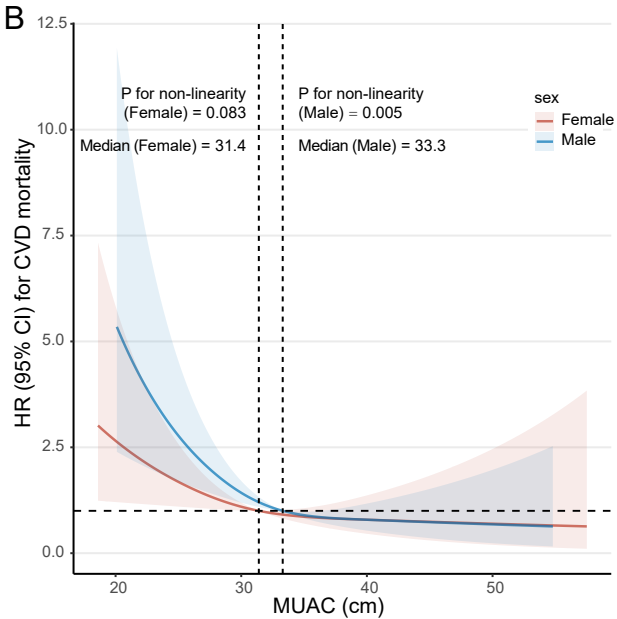**C**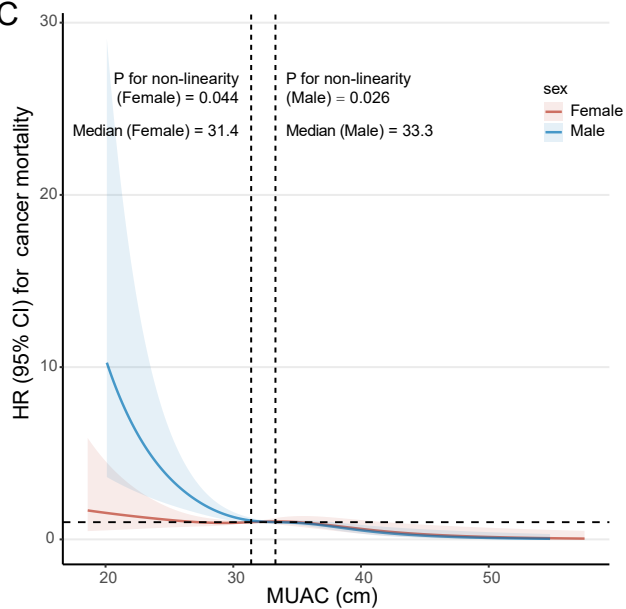**D**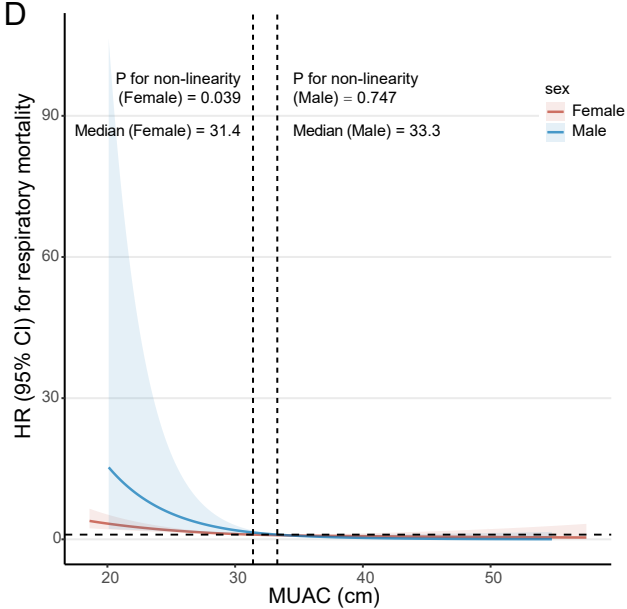

Supplement: Supplementary file 2 — Additional file 2: Figure S2. Survey-weighted restricted cubic spline analyses of the associations of continuous MUAC with all-cause mortality (A), CVD mortality (B), cancer mortality (C), and respiratory mortality (D) in male and female participants. Solid blue and red lines are multivariable-adjusted HR estimations, and the shaded areas are the corresponding 95% CIs. The reference points were set at the median of each subgroup. [file 12889_2023_17064_MOESM2_ESM.pdf]

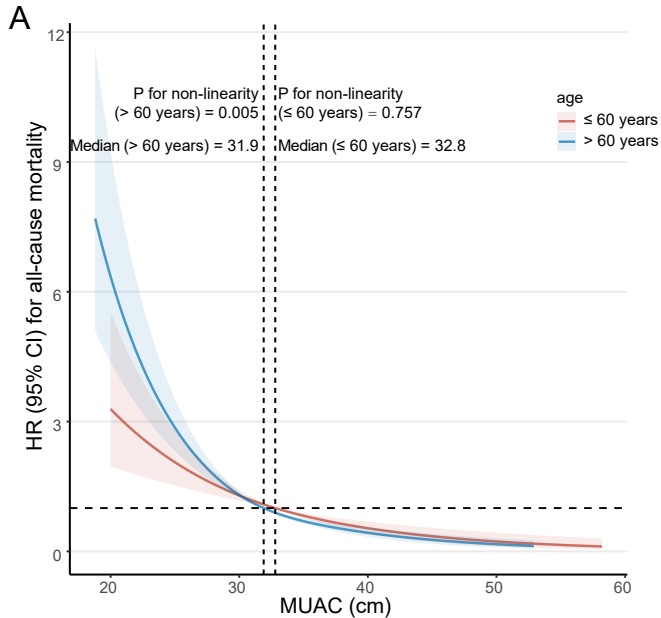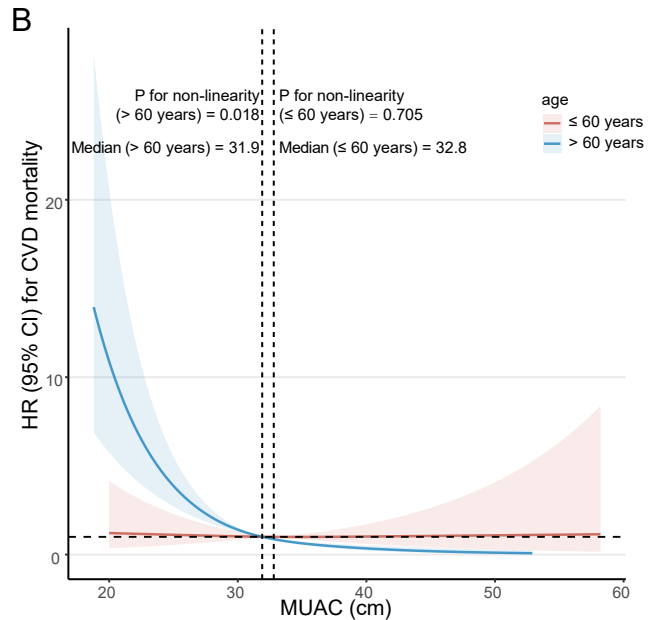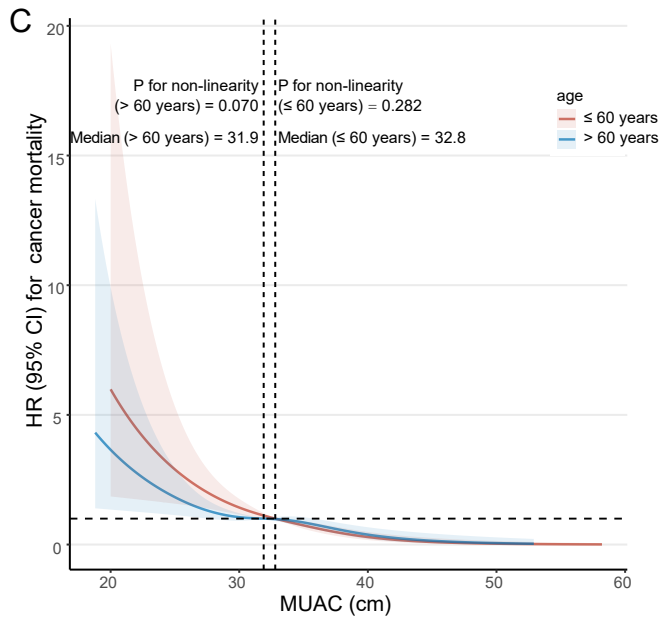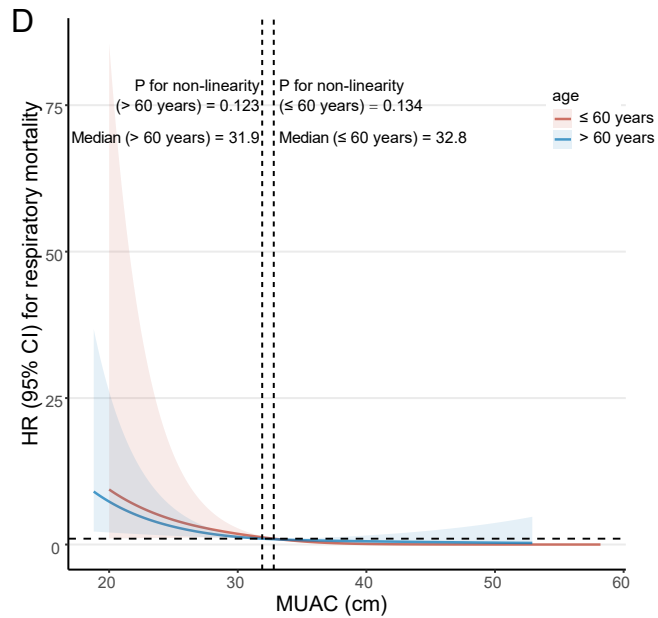

Supplement: Supplementary file 3 — Additional file 3: Figure S3. Survey-weighted restricted cubic spline analyses of the associations of continuous MUAC with all-cause mortality (A), CVD mortality (B), cancer mortality (C), and respiratory mortality (D) in participants with age > 60 and ≤ 60. Solid blue and red lines are multivariable-adjusted HR estimations, and the shaded areas are the corresponding 95% CIs. The reference points were set at the median of each subgroup. [file 12889_2023_17064_MOESM3_ESM.pdf]

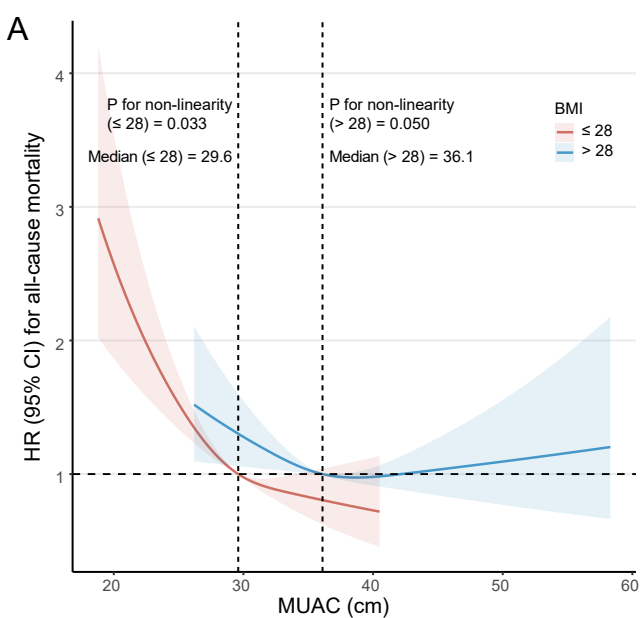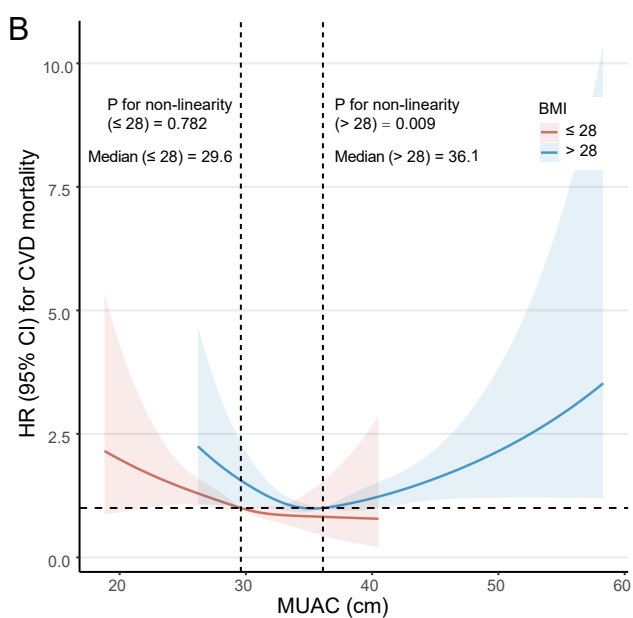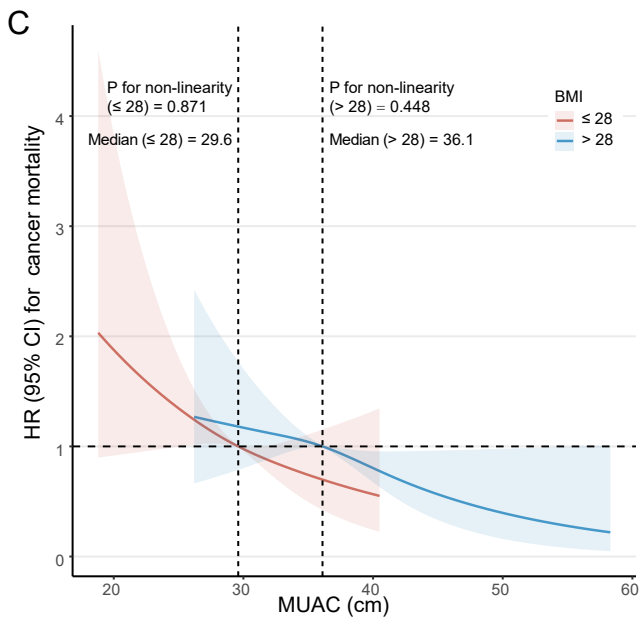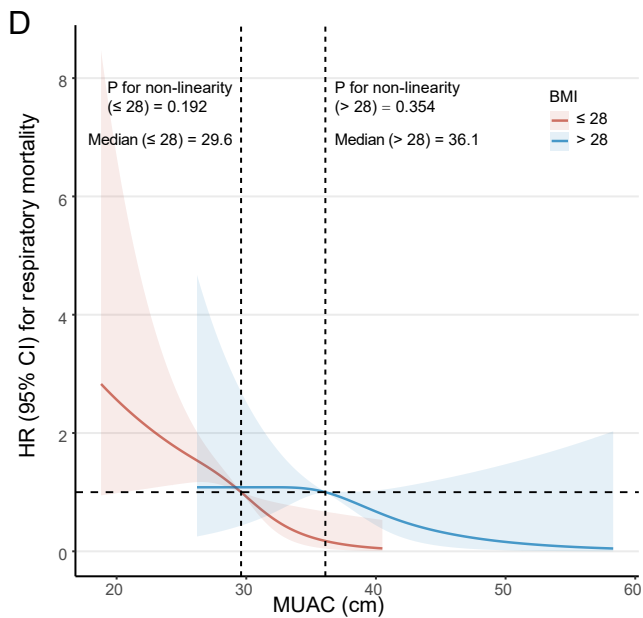

Supplement: Supplementary file 4 — Additional file 4: Figure S4. Survey-weighted restricted cubic spline analyses of the associations of continuous MUAC with all-cause mortality (A), CVD mortality (B), cancer mortality (C), and respiratory mortality (D) in participants with BMI > 28 and ≤ 28. Solid blue and red lines are multivariable-adjusted HR estimations, and the shaded areas are the corresponding 95% CIs. The reference points were set at the median of each subgroup. [file 12889_2023_17064_MOESM4_ESM.pdf]
